# Supplementary material for: Comparative Performance of Citrate, Borohydride, Hydroxylamine and β-Cyclodextrin Silver Sols for Detecting Ibuprofen and Caffeine Pollutants by Means of Surface-Enhanced Raman Spectroscopy
Source: Nanomaterials (Basel). 2020 Nov 25;10(12):2339. doi: 10.3390/nano10122339 (PMC7760587; doi:10.3390/nano10122339)
Supplement: Supplementary file 1 [file nanomaterials-10-02339-s001.pdf]

nanomaterials

MDPI

Supplementary Materials

Comparative Performance of Citrate, Borohydride, Hydroxylamine and β-Cyclodextrin Silver Sols for Detecting Ibuprofen and Caffeine Pollutants by Means of Surface-Enhanced Raman Spectroscopy

Michele Lemos de Souza [1,*], Juan Carlos Otero [2] and Isabel López-Tocón [2,*]

[1] Instituto de Ciências Exatas, Universidade Federal Fluminense, Volta Redonda 27213-145, Rio de Janeiro, Brazil

[2] Andalucía Tech, Unidad Asociada IEM-CSIC, Departamento de Química Física, Facultad de Ciencias, Universidad de Málaga, E-29071 Málaga, Spain; jc_otero@uma.es

* Correspondence: michele_lemos@id.uff.br (M.L.d.S.); tocon@uma.es (I.L.-T.)

Table S1. Pictorial representation and B3LYP/6-31G* optimized structures of the studied molecules.

Trans-cinnamic acid[a] (TCA)	Ibuprofen sodium salt (IBU)[b]	Caffeine (CAF)
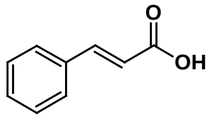	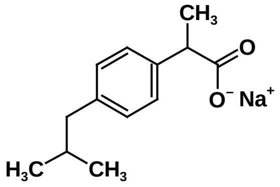	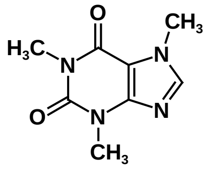
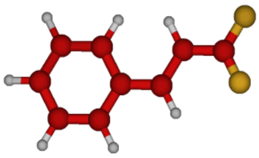	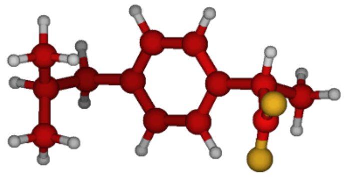	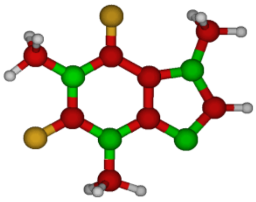

a, b: Optimization of trans-cinnamate and (S)-IBU in the theoretical calculations.

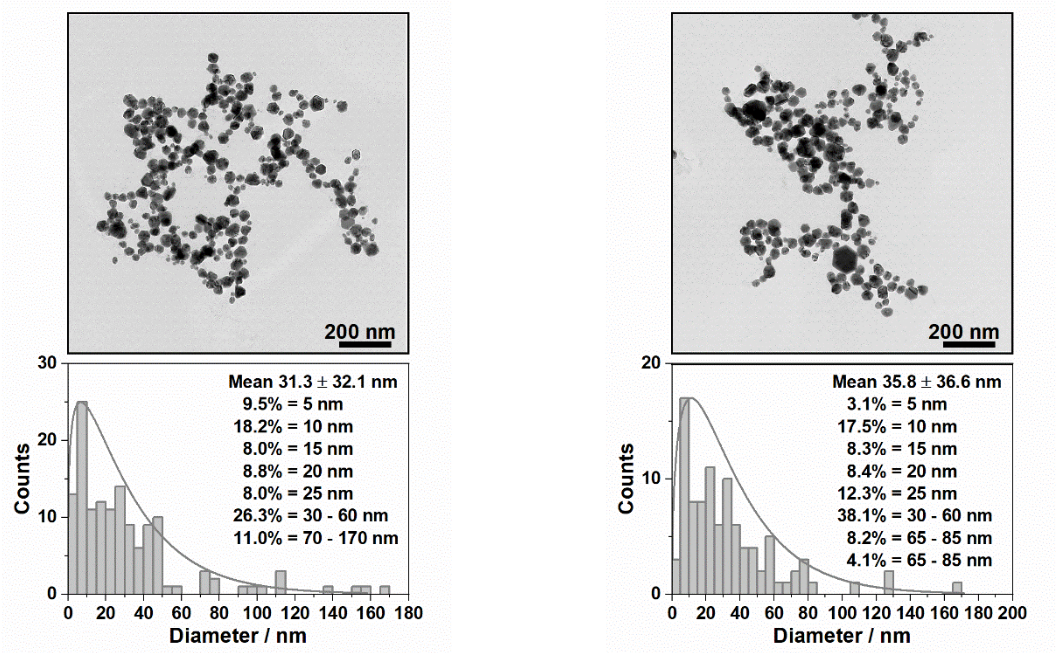

Figure S1. Two TEM images and their respective histograms of Ag@βCD1 NPs.

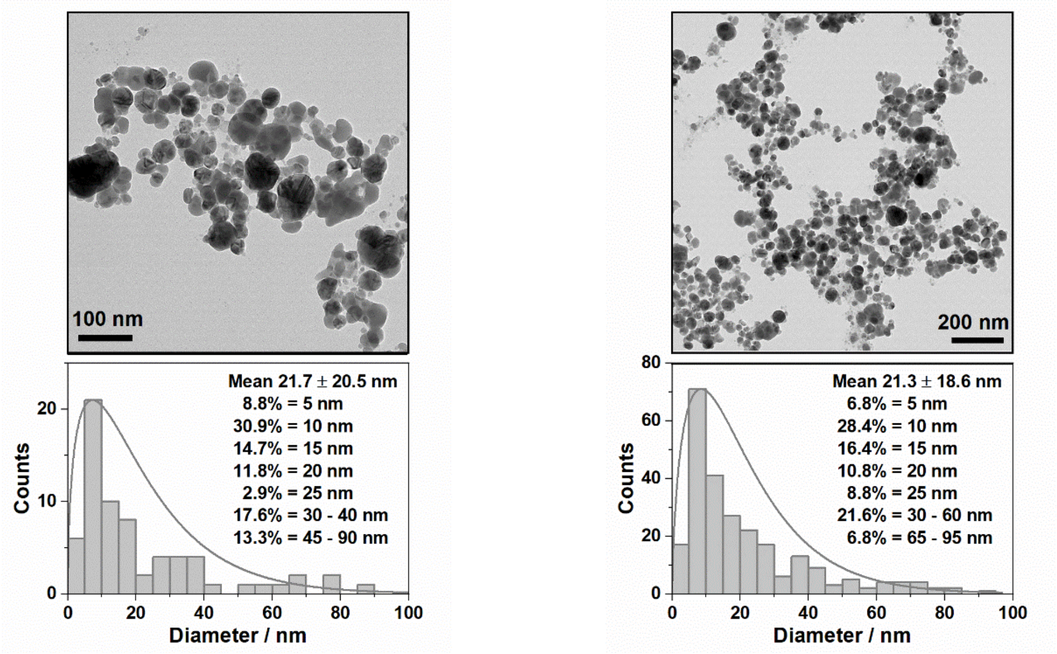

Figure S2. Two TEM images and their respective histograms of Ag@βCD2 NPs.

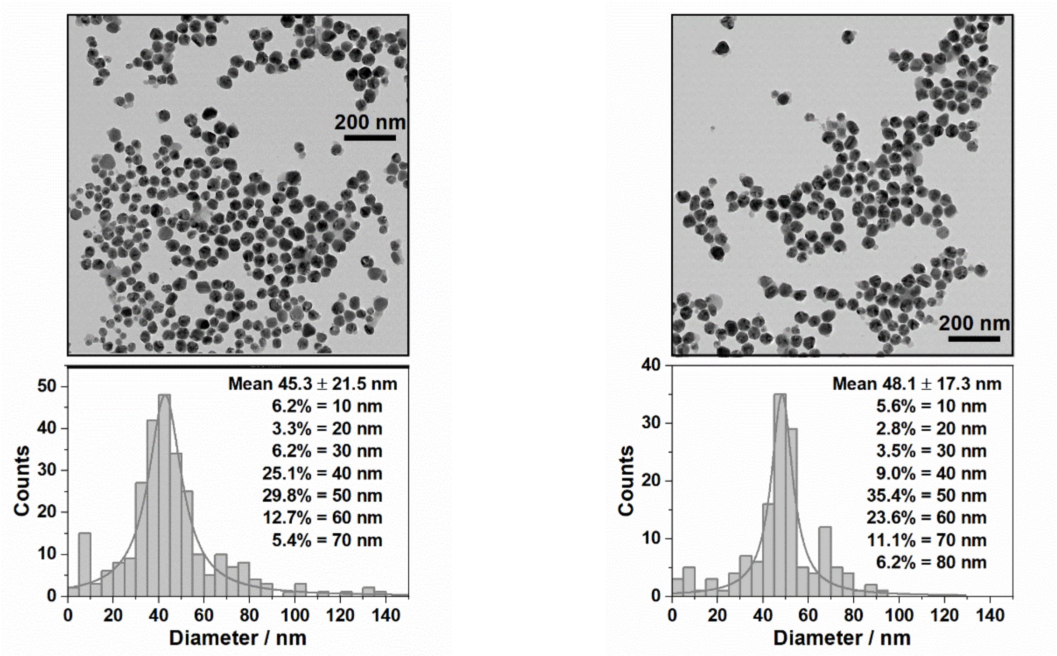

Figure S3. Two TEM images and their respective histograms of Ag@βCD3.

Table S2. Vibrational assignment proposed for the characteristic bands of TCA.

**Raman Solid**	**SERS Ag@BH**	**SERS Ag@HX**	**SERS Ag@Citr**	**SERS Ag@βCD2**	**SERS Ag@βCD3**	**B3LYP[a] 6-31G***	**Assignment[b]**
1636	1635	1636	1636	1634	1635	1676	ν(C=C)
1599	1600	1601	1602	1600	1601	1654	8a;ν_{ring}
			1576	1586		1623	8b;ν_{ring}
1495			1496	1488	1497	1541	19a;ν_{ring}
1442	1450	1447	1449	1422	1449	1490	19b;ν_{ring}
1328	1384	1388	1388	1378	1378	1354	νs(COO)
1291				1290	1291	1314	$\delta(CH)_{et} + \delta(CH)_{bz}$
1265	1253	1253	1253	1251	1254	1259	$\nu(C_{bz}\text{-}C_{et})$+ ν(C-COO)
1211	1205	1206	1204	1204	1207	1216	$\delta(CH)_{et} + \delta(CH)_{bz}$
1177	1182	1182	1182		1180	1200	$\delta(CH)_{et} + \delta(CH)_{bz}$
1160	1161			1162	1162	1183	$\delta(CH)_{bz}$
1000	1000	1002	1002	1002	1002	1012	12;δ_{ring}
				720		723	1;ν_{ring} + δ(COO)
				560		581	6a;δ_{ring} + ν(C-COO)

a: Vibrational wavenumbers of trans-cinnamate. b: Wilson´s nomenclature, ν: stretching, δ: in-plane deformation, bz: benzene, et: ethylene.

Table S3. Vibrational assignment proposed for the characteristic bands of CAF.

**Raman solid**	**SERS Ag@BH**	**SERS Ag@HX**	**SERS Ag@Citr**	**SERS Ag@βCD3**	**B3LYP/ 6-31G***	**Assignment[a]**
1699	1689	1714	1680	1680	1755	ν(C=O) + $\nu(CN)_{Pyrm}$
1607	1606	1605			1641	$\nu(CC)_{Pyrm\text{-}Im}$ + $\nu(CC)_{Pyrm}$
	1360	1363			1370	$\delta(CH_3)$ + $\nu(N\text{-}CH_3)$ + $\nu(CN)_{Pyrm\text{-}Im}$
1337	1330	1324		1329	1396	$\nu(CN)_{Im}$
1242	1251	1238	1252	1254	1269	$\nu(CN)_{Pyrm}$ + $\nu(CH)_{Im}$
1081	1075				1095	$\delta(CH_3)_{Im}$ + $\nu(CN)_{Im}$
1036	1008		1010	1008	1046	$\nu(CN)_{Pyrm}$ + $r(CH_3)_{Pyrm}$ + $\delta(CH)_{Im}$
743	748	745			757	$\delta(CN)_{Im}$ + $\nu(C\text{-}CH_3)_{Im}$
	695	696	695	694	700/737	$\gamma(CH)_{Im}/\tau(CN)_{Pyrm}$
653	647	647	649	649	648	6a;$\delta(CN)_{Pyrm}$
557	507	555		507	557	1;$\nu(CC, CN)_{Pyrm}$

a: Nomenclature, ν: stretching, δ: in-plane deformation, γ: out-of-plane deformation, Pyrm: Pyrimidine, Im: Imidazole. Wilson´s nomenclature for two last normal modes, 6a and 1, according to the benzene-like molecules.

Table S4. Vibrational assignment proposed for the characteristic bands of IBU.

**Raman solid**	**SERS Ag@BH**	**SERS Ag@HX**	**SERS Ag@Citr**	**SERS Ag@βCD3**	**B3LYP/ 6-31G***	**Assignment[a]**
	1652	1652			1759	ν(C=O)
1612	1614	1612	1612	1613	1661	8a;ν_{ring}
1463	1463	1461	1461	1459	1464	19a;ν_{ring} + ν(COO)
	1390	1391	1391		1415	ν(COO) + $\delta(CH_3)$
	1360	1358	1358	1360	1397	$\delta(CH_2)$ + $\delta(CH_3)$
1287	1254	1260	1257	1257	1317	14;ν_{ring} + $\delta(CH)_{chiral}$
1187	1186	1185	1184	1185	1204	$\delta(CH)_{bz}$ + $\nu(C_{bz}\text{-}CH_{chiral})$
		1002	1002		1041	12;δ_{ring}
837	886	887	887	887	900	$r(CH_2)$ + $r(CH_3)$
801	834	834	834	835	841	1;ν_{ring} + $r(CH_3)$
		803	801	801	811	$r(CH_3)$ + γ(COO) + $\gamma(CH)_{bz}$

a: Wilson´s nomenclature, ν: stretching, δ: in-plane deformation, γ: out-of-plane deformation, r: rocking.

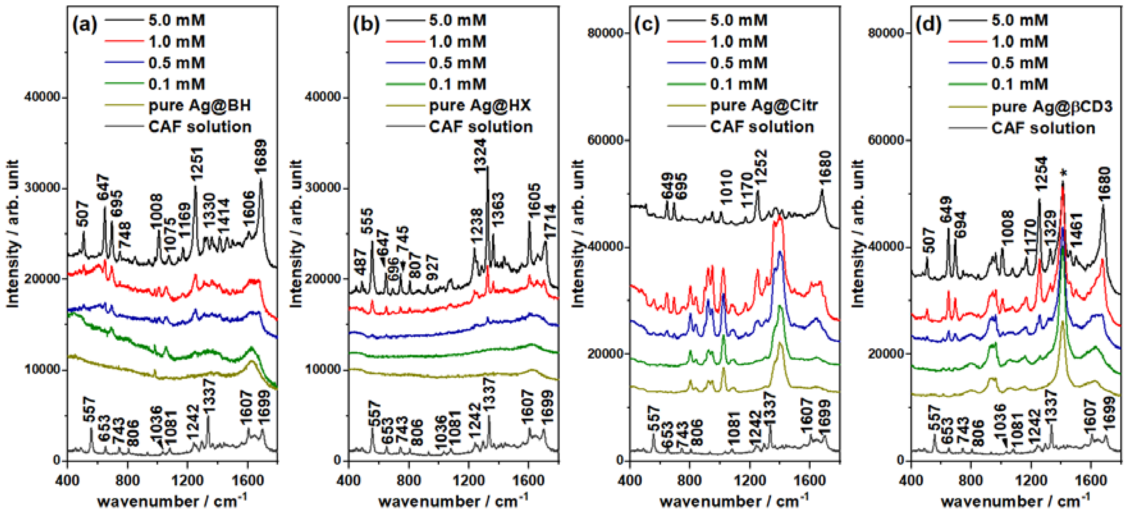

Figure S4. Original SERS spectra of CAF recorded at different concentration and using the NPs (**a**) Ag@BH; (**b**) Ag@HX; (**c**) Ag@Citr and (**d**) Ag@βCD3.

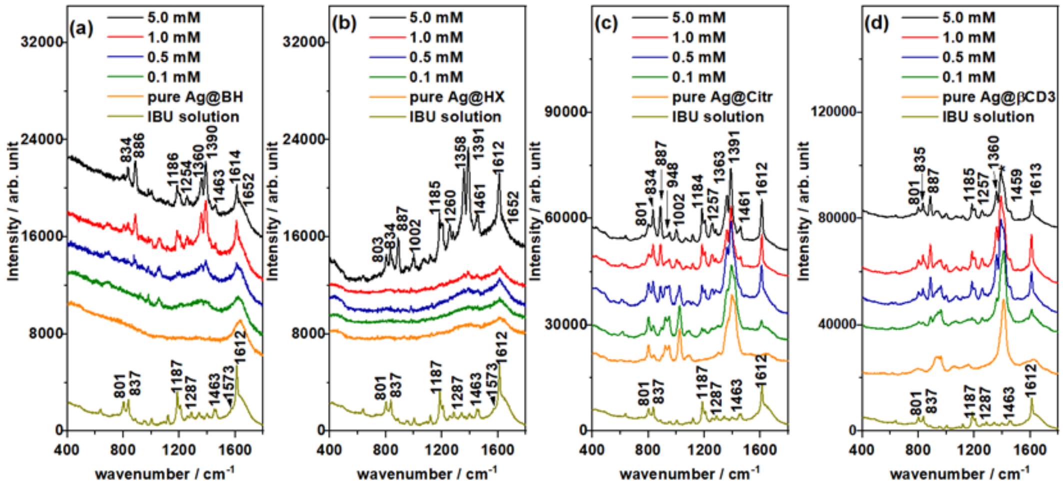

Figure S5. Original SERS spectra of IBU recorded at different concentration and using the NPs (**a**) Ag@BH; (**b**) Ag@HX; (**c**) Ag@Citr and (**d**) Ag@βCD3.
